# Comparison of hospitalist morale in a COVID-19 alternate care site (ACS) to hospitalist morale in conventional hospitals in Maryland

**DOI:** 10.1371/journal.pone.0288981

**Published:** 2023-08-02

**Authors:** Catherine Washburn, Melinda E. Kantsiper, Rogette Esteve, Ishaan Gupta, Gulzeb Memon, Henry J. Michtalik, Bayview Educational and Academic Research (BEAR) Core Consortium

**Affiliations:** 1 Division of Hospital Medicine, Johns Hopkins Bayview Medical Center, Johns Hopkins University, Baltimore, Maryland, United States of America; 2 Baltimore Convention Center Field Hospital, Baltimore, Maryland, United States of America; Alexandria University Faculty of Nursing, EGYPT

## Abstract

**Background:**

Morale and burnout were concerns for hospitalists prior to the COVID-19 pandemic; these concerns were amplified as COVID-19 spread and hospitals experienced unprecedented stress. In contrast to prior literature, our study assesses both satisfaction and the importance of various factors. This study examines morale of hospitalists early in the COVID-19 pandemic in two settings: conventional hospitals and a COVID-19 Alternate Care site (ACS) in the same geographic region in Maryland. Multiple studies published early in the pandemic show low morale in COVID-19 hospitals.

**Methods:**

In a cross-sectional survey study, we analyze data from the Hospitalist Morale Index (HMI) administered between September 2020 and March 2021 to determine the pandemic’s impact on hospitalist morale.

**Results:**

Surprisingly, our study found morale in the ACS was better than morale at the conventional hospitals. ACS hospitalists and conventional hospitalists were demographically similar. Our results show that a significantly higher proportion of conventional hospitalists reported burnout compared to the ACS hospitalists. General quality of life was rated significantly higher in the ACS group than the conventional group. Significantly more ACS hospitalists were invested in making their group outstanding. Five main HMI domains were examined with questions on a 5-point rating scale: Clinical Factors, Workload, Material Rewards, Leadership, and Appreciation/Acknowledgement. ACS hospitalists rated most measures higher than conventional hospitalists; largest differences were observed in Clinical Factors and Appreciation/Acknowledgement domains. Narrative comments from ACS hospitalists revealed strong identification with the mission of the ACS and pride in contributing during a crisis. One key difference between the two groups explains these findings: provider autonomy. The ACS staff chose the position and the assignment, while conventional hospitalists caring for COVID-19 patients could not readily opt out of this work.

**Conclusion:**

Our data suggest that autonomy in assignments with risk has implications for morale and burnout.

## Introduction

Healthcare worker burnout was high prior to the COVID-19 pandemic [1], but the unique confluence of circumstances presented by COVID-19, such as high contagiousness with risk to caregivers and the duration of the pandemic, exacerbated this crisis. The early pandemic stressed healthcare capacity globally with high occupancy of Intensive Care Unit (ICU) beds, and in some locations, adoption of crisis standards of care [2]. The Healthcare Worker Exposure Response and Outcomes (HERO) registry, created to study the impact of the pandemic, surveyed over 55,000 U.S. healthcare workers including physicians [3]. Using HERO registry data, Nieuwsma [4] found rates of potential moral injury, witnessing immoral acts by others, to be similar in COVID-19 healthcare workers and post 9/11 combat veterans. A provider focus-group study captured the breadth and depth of what providers felt they needed from their healthcare institutions: "Hear me," "Protect me," "Support me," "Prepare me," and "Care for me," [5].

Alternate Care Sites (ACSs) were created or re-purposed in some locations to accommodate surges of COVID-19 patients [6]. Some ACSs were temporary hospitals that already existed prior to the pandemic and were not ideally suited to manage patients with contagious respiratory infections [7], some were created at the beginning of the pandemic when personal protective equipment supplies were insufficient [8]. Morale in ACSs has been threatened by long hours [7], fears of infection or infecting loved ones [9], forced separation from family [10], lack of sufficient personal protective equipment, and illness and death of colleagues [9]. Data from COVID-19 hospitals and ACSs show low morale and high rates of insomnia, anxiety and depression in hospitalists and nurses [7, 11, 12]. Studies of morale in healthcare workers during COVID-19 are a fresh area of inquiry. There is a paucity of data comparing morale in different settings and our study adds findings that suggest a given setting’s policies affect clinician morale. We compared morale at the Baltimore Convention Center Field Hospital (BCCFH), a COVID-19 ACS, to regional conventional hospitals using the validated Hospitalist Morale Index (HMI) [13] between September 2020 and March 2021. The HMI is an instrument designed to assess morale, well-being, and burnout. The objective of the present study was to compare hospitalist morale at a COVID-19 ACS to hospitalist morale in conventional hospitals in the same region during the COVID-19 pandemic and identify areas for potential interventions.

## Methods

### Setting

The BCCFH was a Maryland Department of Health COVID-19 ACS jointly operated by Johns Hopkins Medicine and the University of Maryland. It was in continuous operation for inpatient care from April 2020-June 2021, cared for 1,495 COVID-19 admissions, and served as a free public mass testing and vaccination site. It continues in operation today providing monoclonal antibodies and test-to-treat care.

### Participants

We completed a cross-sectional survey of two groups of hospitalists via e-mail link in September 2020 (5 conventional hospitalist programs within a regional healthcare network, n = 183) as scheduled and in March 2021 (BCCFH hospitalists at a single ACS, n = 69) after state and additional approvals. Those working in the BCCFH cared exclusively for patients with COVID-19, admitted directly from emergency departments or from other hospitals, who met specific requirements such as no more frequently than daily blood work, oxygen requirements of ≤4L by nasal cannula, and intermittent, not continuous, intravenous medications. Total assistance/bedbound and patients lacking capacity were excluded on referral. BCCFH clinicians who had performed clinical shifts within the past 3 months were included in this survey. A detailed description of the recruitment and hiring process for the BCCFH has been described elsewhere [14]. Briefly, clinicians were licensed physicians and experienced (minimum two years) advanced practice providers with internal medicine, family practice, emergency medicine, or surgical training. The conventional hospitalists were employed at academic medical centers and community hospitals in the Baltimore region and rotated on both inpatient non-intensive care COVID-19 and non-COVID-19 units. Moonlighters, including those in fellowship or who worked at multiple institutions, were excluded from the survey. Consent to complete the survey was completed online prior to entering the survey. This study was reviewed and approved by the Johns Hopkins University Institution Review Board (IRB00249880) on June 25, 2020.

### Survey instrument

The survey instrument was the validated Hospitalist Morale Index (HMI), which assesses self-reported quality of life, burnout (emotional exhaustion and/or depersonalization) [15], commitment to group excellence and thoughts of leaving the current group. The HMI is comprised of 5 main domains (Clinical factors, Workload, Material Rewards, Leadership, and Appreciation/Acknowledgement), each with several subdomains, and 5 single item indicators (Overall culture, Job security, Autonomy/Independence, Work/life balance and Professional growth opportunities). Overall, domain and single item indicator scores are weighted means of items based on importance and satisfaction ratings, ranging from 0 (low) to 5 (high). Demographic factors surveyed included sex, race/ethnicity, and age. Clinical factors included provider academic rank or position and years as a hospitalist. The survey also included an opportunity to provide narrative comments.

Using a convenience sample, the HMI was administered over a 3-week period, with reminders two times a week with alternating weekend days targeted to those who did not respond. The confidential survey with personalized links and reminders was distributed via email using Qualtrics^XM®^ software. Personalized links can only be completed once to prevent multiple responses. Once the survey was closed, any identifiers were removed before analysis.

### Statistical analysis

Statistical analyses employing t-tests, chi-squared tests, and ANOVA examined differences in continuous and categorical factors, and HMI respectively, between the conventional and BCCFH hospitalist groups (STATA^®^13).

## Results

Of the 69 BCCFH hospitalists, 55 (80%) responded to the survey; of the 183 conventional hospitalists, 141 (77%) responded. Demographically, the hospitalist groups were similar, apart from ethnicity (Table 1), which varied significantly. More BCCFH than conventional hospitalists were African American (30% versus 5% respectively, *p*<0.01). Fewer BCCFH than conventional hospitalists were Asian (21% versus 42% respectively, *p*<0.01). Caucasian and Other categories were similar at both sites. Provider academic rank or position differed significantly, with fewer faculty physicians and more nurse practitioners/physician assistants at the BCCFH. Forty-five percent of BCCFH hospitalists were nonfaculty physicians, 9% faculty physicians and 45% NPPAs, versus 51% nonfaculty physicians, 38% faculty physicians and 11% nurse practitioner/physician assistants for conventional hospitalists (*p*<0.001).

**Table 1:** Clinician demographics. Baltimore Convention Center Field Hospital (BCCFH) Versus Conventional Hospitalists.

Variable	BCCFH Hospitalists (n = 55)	Conventional Hospitalists (n = 141)	*p* value
Sex, n (%)			0.56
Male	23 (42)	66 (47)	
Female	32 (58)	75 (53)	
Ethnicity, n (%)			<0.01
Caucasian	20 (38)	52 (38)	
African American	16 (30)	7 (5)	
Asian	11 (21)	57 (42)	
Other	6 (11)	21 (15)	
Age (years), n (%)			0.12
25–34	10 (19)	37 (27)	
35–44	20 (38)	63 (45)	
45–64	23 (43)	39 (28)	
Provider academic rank or position, n (%)			<0.001
Nonfaculty physician	25 (45)	72 (51)	
Faculty physician	5 (9)	53 (38)	
Nurse practitioner/physician assistant	25 (45)	16 (11)	
Duration of hospitalist career (years), n (%)			0.24
≤6	22 (40)	69 (49)	
≥7	33 (60)	71 (51)	

Measurements of self-reported burnout, with subcategories of emotional exhaustion and depersonalization, quality of life, morale as a hospitalist in current group, thoughts of leaving the current group and commitment to making the group outstanding differed significantly between the two study populations (Table 2). Four percent of BCCFH hospitalists versus 23% of conventional hospitalists endorsed burnout (*p*<0.01), which was defined as emotional exhaustion and/or depersonalization occurring once a week or more. Four percent of BCCFH hospitalists experienced emotional exhaustion weekly or more often, compared to 20% of conventional hospitalists (*p*<0.01). Depersonalization experienced weekly or more often was endorsed by none of BCCFH hospitalists versus 10% of the conventional hospitalists (*p* = 0.01). Significantly more BCCFH than conventional hospitalists reported good quality of life, 91% versus 70% (*p*<0.01). Quality of life in the HMI was defined: as good as it can be, or good, versus okay, somewhat bad, or as bad as it can be. Self-reported “morale as a hospitalist in my current group,” the top three quintiles (excellent, very good or good versus fair or poor), was endorsed by 95% of BCCFH hospitalists versus 74% of conventional hospitalists (*p*<0.01). “Morale of my current group,” defined as excellent, very good or good versus fair or poor, was rated higher at BCCFH than at conventional hospitals but did not reach statistical significance (85% versus 74%, *p* = 0.09). Likewise, for the category “recommend my current group as great to work with,” the top two quartiles (absolutely or yes versus no or definitely no) were chosen by 98% of BCCFH hospitalists versus 92% of conventional hospitalists (*p* = 0.19). Significantly fewer BCCFH than conventional hospitalists had serious thoughts of leaving their group, defined as absolutely or yes versus no or definitely no because they were unhappy (13% versus 28%, *p* = 0.04). “Commitment to making my hospitalist group outstanding,” was highly rated, defined as tremendously or quite a lot versus somewhat, a little or not at all, by 91% of BCCFH hospitalists, versus 67% of conventional hospitalists (*p*<0.01).

**Table 2:** Self-reported morale. BCCFH Versus Conventional Hospitalists.

Self-Reported Variable	BCCFH Hospitalists, n (%)	Conventional Hospitalists, n (%)	*p* value
Overall burnout	2 (4)	32 (23)	<0.01
Emotional exhaustion	2 (4)	28 (20)	<0.01
Depersonalization	0 (0)	14 (10)	0.01
Good quality of life	50 (91)	99 (70)	<0.01
Morale as hospitalist in current group	52 (95)	104 (74)	<0.01
Morale of my current group	47 (85)	104 (74)	0.09
Recommend my group as great to work with	54 (98)	130 (92)	0.19
Serious thoughts of leaving the group	7 (13)	39 (28)	0.04
Commitment to making my hospitalist group outstanding	50 (91)	94 (67)	<0.01

As part of the HMI score, 5 main domains and 5 single item indicators were examined on a 5-point scale (Table 3). In the Clinical Factors domain, BCCFH hospitalists rated ratio of face time to documentation significantly higher than conventional hospitalists (3.10 versus 2.36, *p*<0.001), but rated relationship with consultants significantly lower (2.16 versus 2.61, *p* = 0.03). In the Workload domain, BCCFH hospitalists rated percentage of night shifts, daily patient census and total shifts per schedule block more negatively than conventional hospitalists (1.15 versus 2.63, *p*<0.001; 2.01 versus 3.14, *p*<0.001; 2.06 versus 3.09, *p*<0.001, respectively). The comments show that BCCFH survey respondents wanted more total shifts and more night shifts, given the compensation model, rather than concerns for excessive workload. In the Material Rewards domain, BCCFH hospitalists ranked pay better than conventional hospitalists (3.17 versus 2.18, *p*<0.001), and benefits worse (2.04 versus 2.45, *p* = 0.04). In the Leadership domain subcategories of leadership fairness, effectiveness, approachability, and availability, amongst others, BCCFH and conventional hospitalists ranked their leadership similarly, although BCCFH hospitalist rankings were slightly higher, but not statistically significantly different. In the Appreciation/Acknowledgement domain, BCCFH again outranked conventional hospitalists, most clearly in the subdomain of “feeling valued in your organization” (3.25 versus 2.61, respectively, *p* = <0.01). For single item indicators, the only statistically significant difference was lower job security for BCCFH compared with conventional hospitalists (2.33 versus 3.41 respectively, *p*<0.001). Narrative comments from both sites directly relating to morale are shown in Table 4 and show strong site clinician identification with the mission of the BCCFH.

**Table 3:** Ratings of Hospitalist Morale Index (HMI) domains. BCCFH Versus Conventional Hospitalists.

Domains		BCCFH Hospitalists (n = 55)	Conventional Hospitalists (n = 141)	
	Items	Mean HMI (±SD)		*p* value
**Clinical Factors**	**Overall**	2.80 (±1.09)	2.64 (±0.81)	0.28
	Variety of cases	2.17 (±1.37)	2.45 (±1.09)	0.13
	ntellectual stimulation	3.16 (±1.31)	2.83 (±1.15)	0.08
	Relationship with patients	3.39 (±1.37)	3.10 (±1.14)	0.13
	Patient face time versus documentation	3.10 (1.41)	2.36 (±1.05)	<0.001
	Relationship with consultants	2.16 (±1.61)	2.61 (±1.12)	0.03
	EMR	2.81 (±1.62)	2.51 (±1.19)	0.16
**Workload**	**Overall**	1.74 (±1.28)	2.95 (±1.17)	<0.001
	Percentage of nights per block	1.15 (±1.42)	2.63 (±1.70)	<0.001
	Daily patient census	2.01 (±1.41)	3.14 (±1.12)	<0.001
	Total number of shifts per block	2.06 (±1.46)	3.09 (±1.28)	<0.001
**Material Rewards**	**Overall**	2.60 (±1.37)	2.31 (±0.92)	0.09
	Pay	3.17 (±1.38)	2.18 (±1.02)	<0.001
	Benefits	2.04 (±1.71)	2.45 (±1.03)	0.04
**Leadership**	**Overall**	3.55 (±1.21)	3.38 (±1.03)	0.32
	Fairness	3.44 (±1.42)	3.45 (±1.18)	0.95
	Effectiveness	3.52 (±1.29)	3.32 (±1.12)	0.29
	Approachability	3.91 (±1.29)	3.67 (±1.12)	0.20
	Availability	3.80 (±1.21)	3.63 (±1.09)	0.32
	Advocates for my needs	3.38 (±1.37)	3.23 (±1.24)	0.47
	Receptiveness to my thoughts and suggestions	3.47 (±1.42)	3.30 (±1.21)	0.40
	Alignment of group’s goals with mine	3.35 (±1.35)	3.07 (±1.21)	0.16
**Appreciation/Acknowledgement**	**Overall**	3.18 (±1.44)	2.84 (±1.04)	0.06
	Receiving feedback from group	3.12 (±1.53)	2.87 (±1.18)	0.22
	Receiving recognition from group	3.00 (±1.58)	2.74 (±1.21)	0.23
	Feeling valued in group	3.35 (±1.50)	3.12 (±1.18)	0.25
	Feeling valued in organization	3.25 (±1.54)	2.61 (±1.21)	<0.01
**Single items**	Culture	3.42 (±1.42)	3.22 (±1.10)	0.31
	Job security	2.33 (±1.42)	3.41 (±1.12)	<0.001
	Autonomy/independence	3.28 (±1.14)	3.16 (±1.15)	0.52
	Work/life balance	3.29 (±1.22)	3.23 (±1.17)	0.76
	Professional growth opportunities	2.73 (±1.35)	2.77 (±1.18)	0.86

Abbreviation: SD = Standard Deviation

**Table 4:** Selected narrative comments. Related to Morale in the HMI Survey.

BCCFH Hospitalists	Conventional Hospitalists
Work that is not just medical care but is also service to a national crisis has a luster about it that will be hard to reproduce. I am worried about where to look next for a position that would provide fulfillment.	There is a balance between work, PPE, and burnout. For example, increasing the census and decreasing providers to save PPE, in turn increases the work without a concomitant decrease in shifts. This strategy in turn increases burnout. Similarly, we "are in it together" should mean the burdens are shared across ALL services, house staff and consultants. Instead, the hospitalists are seen as a "fixer" with limited or soft caps, while other services get to say no or have hard caps. It is simply not fair.
The feeling of teamwork-from all team members at all levels-has made it impossible to not have high morale at times.	External factors from COVID-19 epidemic have a big impact on work-life balance. In many ways it is easier to let work intrude into person time because there is such a vacuum. In otherwise there is a general disconnection between coworkers, consultants, staff due to social distancing. Tough therefore to make clear judgements about "the group" with such confounders.
I am very proud of my work at the field hospital. I was one of the first physicians on board and I found the leadership extremely receptive to ideas from all participants. I loved being on the cutting edge as we all navigated caring for patients with the novel Coronavirus. The team truly lived up to the motto “One Team One Fight.” I found it an honor to have practiced there.	The frenetic pace of change in response to the global pandemic has been exhaustingly challenging this year. Obviously harder on nurses, techs, environmental staff, and others on the wards, but hard on docs too. Some decisions made by the school of medicine regarding benefits are particularly odious (e.g., suspension of retirement contributions, barriers to utilization of CME accounts), particularly in the unilateral process made in their implementation. I’ve certainly lost some enthusiasm for and trust in my employer as a consequence. Nevertheless, I feel incredibly lucky to have the job I have and the colleagues with whom I work.
The BCCFH is a unique place to work as providers come from so many other hospitals, specializations. In general, they are good providers with amazing personality and respect to each other. I have enjoyed working in the field hospital and made nice friends. I am very happy that I decided to help with the COVID-19 patients and learned valuable lessons.	During COVID pandemic our group came together and worked very hard to keep our hospital afloat. I feel administration has forgotten this and can’t even give us a decent office or bonus we deserve/earned. This impacts morale more than anything.
I enjoyed working in the field hospital and thank you for those who are on the leadership position to help and support us in every corner to make sure we are okay and healthy.	Great leaders in our group, very much appreciate their efforts. They create a good / supportive work environment, proud to be a part of their team.
Great group of folks to work with and the organization was flexible with policy adapting as needed to ensure safety and best patient practices.	The group would benefit from spending more time on making people feel valued as people.

Note: Each comment is from a single respondent and no respondent commented more than once.

## Discussion

Our study compares morale in a COVID-19 ACS to that in conventional hospitals and reports higher morale among providers at the BCCFH ACS compared to hospitalists at conventional hospitals. This contrasts with other studies which have reported low morale at other COVID-19 ACSs [9]. Other studies have shown high rates of burnout [12] anxiety [11] and insomnia [9] in many providers caring for COVID-19 patients in ACSs and elsewhere early in the pandemic. Given the low morale widely reported in other COVID-19 ACSs, our finding of significantly higher morale in the BCCFH, compared with local conventional hospitals, is unexpected. Morale is a concept that captures not only job satisfaction, but also contentment and happiness [13]. In contrast to prior literature, our study assesses both satisfaction and the importance of each of these domains. Future studies should explore how to increase clinicians’ feelings of being valued in an organization, increase face time with patients, reduce administrative burden, and engender autonomy, which have all been shown to be improve morale. Specific measures that have been used in our hospital medicine group to build morale during the pandemic include frequent update meetings, respite stations in COVID-19 units, remote social activities and “shoutouts” to highlight individual accomplishments. At the BCCFH, the sense of mission was important. The field hospital gave providers a way to respond to a crisis in a hands-on way. They were excited to build new things, such as new service lines (M. E. Kantsiper, personal communication April 11, 2022). We believe high morale resulted from the staff’s strong identification with the mission of the BCCFH, as shown in many of the open-ended survey comments, and from their being a self-selected group from a pool of local providers. Whereas conventional hospitals were struggling to increase their capacity and asking their hospitalists to do more and different activities from their usual work, this ACS was specifically set up to treat COVID-19 patients.

A key difference between BCCFH and conventional hospitalists was in freedom of choice to care for COVID-19 patients. The COVID-19 ACSs described in earlier studies were staffed by individuals that may not have chosen the assignment [10] and similarly, for conventional hospitalists, there was no choice as to whether to care for COVID-19 patients. Significant differences between the two groups were observed in the Clinical, Workload, and Appreciation/Acknowledgement domains, and in self-rated measures of burnout, professional satisfaction, and quality of life. Notably, the only employment benefit for BCCFH hospitalists was paid leave in case of infection with COVID-19, yet the respondents rated benefits as nearly as highly as conventional hospitalists who had full employment benefits. The unique structure and environment of the BCCFH led to a higher ratio of face time to documentation and to more hospitalists feeling valued in their organization. Future studies should explore how to increase clinicians’ sense of being valued by their organization and how to increase face time with patients which have been shown to be indicators of hospitalist morale, in addition to exploring ways to minimize administrative burden on clinicians.

Potential study limitations include that it is a single ACS site survey and that the conventional hospitalists were surveyed in the fall of 2020 when a vaccine was not yet available, and the ACS hospitalists were surveyed just after. While vaccination timing may have boosted overall morale in the ACS group, we do not believe it explains all the differences between the two groups in all the domains surveyed, and no one commented on vaccination in the open-ended comments. Also, studies have shown providers have experienced significant mental health symptoms during the pandemic in conventional hospitals, and this may have mitigated any potential differences [4, 16]. Differences in the patient populations of the sites may have played a small role in morale, however patient acuity was not mentioned in providers’ comments from either site. None of the hospitalists provided ICU level of care. The response rates for BCCFH and conventional hospitalists were 80% and 77% respectively, exceeding the suggested threshold of 60%, and minimizing concerns for non-response bias [17]; review of respondent demographics with site leadership found the data to be representative of the individual sites.

This study shows that caring for COVID-19 patients per se does not necessarily produce low morale; even in a COVID-19 ACS, hospitalists were highly engaged in this mission and few reported burnout. This survey demonstrates the impact of face time with patients, feeling valued in one’s organization, and compensation on hospitalist morale. Because autonomy and freedom of choice are drivers of provider morale and satisfaction, future policies in hospitalist staffing should consider individual clinicians’ desire to contribute to new initiatives and prioritize autonomy in assignments that involve risk.
